# Next-Generation Sequencing Comparative Analysis of DNA Mutations between Blood-Derived Extracellular Vesicles and Matched Cancer Tissue in Patients with Grade 4 Glioblastoma

**DOI:** 10.3390/biomedicines10102590

**Published:** 2022-10-15

**Authors:** Paolo Rosa, Elena De Falco, Luca Pacini, Amedeo Piazza, Paolo Ciracì, Luca Ricciardi, Francesco Fiorentino, Sokol Trungu, Massimo Miscusi, Antonino Raco, Antonella Calogero

**Affiliations:** 1 Department of Medical-Surgical Sciences and Biotechnologies, Sapienza University of Rome, C.so Della Repubblica 79, 04100 Latina, Italy; 2 Mediterranea Cardiocentro, 80122 Naples, Italy; 3 Operative Unit of Neurosurgery, Department of NESMOS, Sapienza University of Rome, 00185 Rome, Italy; 4 Department of Molecular Medicine, Sapienza University of Rome, 00185 Rome, Italy; 5 UO di Neurochirurgia, Azienda Ospedaliera Cardinal G. Panico, 73039 Tricase, Italy

**Keywords:** glioblastoma, extracellular vesicles, next-generation sequencing, pathogenic mutations, NF1

## Abstract

The biological heterogeneity of glioblastoma, IDH-wildtype (GBM, CNS WHO grade 4), the most aggressive type of brain cancer, is a critical hallmark, caused by changes in the genomic mutational asset and influencing clinical progression over time. The understanding and monitoring of the mutational profile is important not only to reveal novel therapeutic targets in this set of patients, but also to ameliorate the clinical stratification of subjects and the prognostic significance. As neurosurgery represents the primary technique to manage GBM, it is of utmost importance to optimize alternative and less invasive methods to monitor the dynamic mutation profile of these patients. Extracellular vesicles (EVs) are included in the liquid biopsy analysis and have emerged as the biological mirror of escaping and surviving mechanisms by many tumors, including glioblastoma. Very few studies have investigated the technical feasibility to detect and analyze the genomic profile by Next-Generation Sequencing (UMI system) in circulating EVs of patients with grade IV glioblastoma. Here, we attempted to characterize and to compare the corresponding matched tissue samples and potential variants with pathogenic significance of the DNA contained in peripheral-blood-derived EVs. The NGS analysis has revealed that patients with grade IV glioblastoma exhibited lesser DNA content in EVs than controls and that, both in EVs and matched cancer tissues, the NF1 gene was consistently mutated in all patients, with the c.2568C>G as the most common pathogenic variant expressed. This study supports the clinical utility of circulating EVs in glioblastoma as an eligible tool for personalized medicine.

## 1. Introduction

Gliomas are brain tumors of glial origin, with about 100,000 new diagnoses every year worldwide [1]. This is the most frequent malignancy of the brain and is associated with a dismal prognosis and quality of life [2]. Gliomas are generally diagnosed by clinical assessment and imaging evaluations, and histological analysis which is mandatory to confirm the diagnosis [3]. In the last decade, this approach has made it possible to distinguish between low (WHO grades I, II, III) and high grades of glioma (glioblastoma, GBM, WHO grade IV), the latter of which is considered the most aggressive form. Despite the best treatments, which are still based on a combination of surgery, radio- and chemotherapy with temozolomide [4], GBM is still an untreatable tumor with a median survival of about 12–15 months [3]. In 2016, the World Health Organization (WHO) suggested integrating the mutational analysis of the isocitrate dehydrogenase gene (IDH1) into the glioma classification, therefore improving tumor diagnostics and patient prognosis [5]. This classification was recently updated in the fifth edition of the WHO classification of tumors of the Central Nervous System (CNS5), and the novel nomenclature for GBM is glioblastoma, IDH-wildtype (CNS WHO grade 4). In order to provide powerful diagnostic and/or prognostic information, TERT promoter mutations, EGFR amplifications, and chromosome 7 gain/chromosome 10 loss were added as molecular biomarkers [6,7]. Nevertheless, the early diagnosis and, in particular, the possibility of monitoring the progression and the molecular rearrangement of the GBM without resorting to invasive procedures is clearly more complex compared to other types of tumors.

Extracellular vesicles (EVs) have been found in biofluids and represent an intercellular mechanism of biomolecule transport [8,9], including by lipids [10], proteins [11], and nucleic acids [12]. Glioblastoma cells release EVs in the local microenvironment and can be detected in the bloodstream (they are able to cross the blood–brain barrier, which is often compromised in these patients), mediating angiogenesis, proliferation, immunomodulation, carcinogenesis, and invasiveness [13,14,15]. Importantly, EVs are acknowledged as useful to following the molecular profile of brain tumors over the time, as the molecular cargo of EVs in patients with GBM can foster not only specific miRNAs and mRNAs, but also a defined genomic signature of the corresponding parental cancer tissue. This property may include the recent observation regarding the extrachromosomal DNA [16], potentially responsible for the molecular heterogeneity and further complexity of GBM. Genomic mutations have been extensively found in circulating DNA and from EVs extracted from cerebrospinal fluid [17,18], which is an invasive procedure for GBM patients, who normally display high intracranial pressure. Thus, the employment of peripheral-blood-derived EV could be more useful to patients with GBM in order to strictly monitor the evolution of the disease.

Studies regarding genomic mutations in the EVs originating from the blood of patients with grade IV GBM are lacking in the literature, except for those regarding in vitro GBM-cell-derived EVs [19,20,21], the characterization of both EVs, and circulating free DNA of blood origin [22,23].

Here, we first compared the genomic mutational profile of peripheral-blood-derived EV from patients with grade IV GBM with the matched cancer tissues by Next-Generation Sequencing (NGS) analysis. We found a high correlation in the mutational status of the NF1 gene, which was mainly represented in both types of samples.

## 2. Materials and Methods

### 2.1. Study Design

The presented investigation consisted of a perspective observational study, conducted at a single academic institution. This study, entitled “Circulating Exosomal-DNA in glioma patients,” was approved by the Council of the Medical Surgical Sciences and Biotechnology Department in 2019, department project number 95. A properly designed informed consent, consistently matching institutional guidelines for scientific investigations, was collected from patients. The presented study accords with the WMA Helsinki declaration of Human Rights. The time range for conducting the presented investigation was set from January 2019 through June 2019, consisting of 6 months.

### 2.2. Inclusion and Exclusion Criteria

Patient group: consecutive patients admitted at our tertiary hospital for brain tumors were considered for eligibility. Pre-operative Karnofsky performance status (KPS) > 70%, total-body contrast-enhanced Computed Tomography (CT) scan negative for solid tumors, and pathological diagnosis of GBM (WHO-2016) were considered as inclusion criteria. Patients underwent brain Magnetic Resonance Imaging (MRI) and had blood samples withdrawn for non-specific cephalgia, which were employed as controls at our Neurologic Department. Images were acquired anonymously by using the institutional Picture Archiving and Communicating System (PACS) viewer in line with standard procedures of the hospital. Subjects were considered for inclusion in the control group in cases where their brain MRI for intracranial solid tumors were negative. The KPS was assessed pre-operatively and before hospital discharge. Complications, recurrence-free survival, and overall survival were registered for every patient.

### 2.3. Radiologic Assessment

A gadolinium-enhanced MRI (gh-MRI) and a non-gh-MRI were performed in patients and controls groups, respectively, and according to the standardized protocol for glioma-and-neurodegenerative diseases at the Neuroradiology Department.

In the patients’ group, gh-t1-weighted and fluid-attenuated inversion recovery (FLAIR) sequences were processed for calculating the volume of the nodular lesion and the perilesional edema, respectively. The grade of resection was calculated on post-operative gadolinium-enhanced MRI (gh-MRI), and rated as gross-total removal, partial removal, or excisional biopsy. A single senior Neuroradiologist personally evaluated images, using the workstation connected to the Picture Archiving and Communicating System (PACS) and the institutional certified PACS viewer.

### 2.4. Tissue Sampling and Histopathologic Analysis

During surgery, tumor samples were collected and fixed in 4% buffered formaldehyde before being embedded in paraffin. Sections of 2µm were stained with a fully automated staining system (Leica Microsystems) for Hematoxylin and Eosin. The sections were also incubated with mouse monoclonal antibodies against human GFAP, OLIG2, ATRX, EGFR, IDH1^R132H^, and Ki67. Diagnosis of certainty was performed according to the World Health Organization Gliomas scheme within 3 weeks of surgery.

### 2.5. Tissue Processing and DNA Extraction

Tumor areas were circumscribed under a light microscope on stained Hematoxylin and Eosin (H&E) slide sections and manually macrodissected from 10 µm unstained Formalin-Fixed Paraffin-Embedded (FFPE) sections prior to DNA extraction with the QIAamp DNA Mini Kit (Qiagen, Hilden, Germany), according to the manufacturer’s protocol. DNA concentration was measured with the Qbit4 (Invitrogen, Waltham, MA, United States). DNA samples with concentrations higher than 2 ng/µL received the approval to be processed for NGS analysis.

### 2.6. Plasma Separation, EV Isolation and DNA Extraction

Whole blood samples from 26 glioma patients (24 h before surgery) and 10 healthy donors were collected in PAXgene blood ccfDNA collection tubes (Qiagen) and centrifuged at 2000× *g* for 10 min at 4 °C within 4 h. Then, the separated plasma was collected and centrifuged at 3000× *g* for 15 min at 4 °C prior to storage at −80 °C. EVs were isolated from blood plasma with the exoEasy maxi kit (Qiagen) following the manufacturer’s instructions. DNA was extracted from the isolated EVs by the QIAamp MinElute Virus spin Kit (Qiagen) according to the manufacturer’s instructions. Due to the lower yield of DNA extracted from GBM plasma EV, we considered suitable for our analysis only those samples with a DNA concentration above 0.5 ng/µL.

### 2.7. Next-Generation Sequencing Analysis

Next-Generation Sequencing analysis was performed with the GeneReader instrument (Qiagen) [24,25] considering a custom U.M.I. (Unique Molecular Indexes) panel, the GeneRead^®^ QIAact GliomaProject DNA Panel CP153 (Qiagen), which considers the following genes: ATRX (NM_000489.4 NP_000480.3), CDKN2A (NM_000077.4 NP_000068.1), H3F3A (NM_002107.4 NP_002098.1), IDH1 (NM_001282386.1 NP_001269315.1), IDH2 (NM_002168.3 NP_002159.2), NF1 (NM_000267.3 NP_000258.1), PTEN (NM_000314.6 NP_000305.3), TERT (NM_198253.2 NP_937983.2), and TP53 (NM_000546.5 NP_000537.3). The UMI system employs a cut-off based on variant allele frequency of 0.5% and a 200x UMI coverage. The minimum mapped reads per library was (millions) 5.33M. Results were analyzed with the QCI-A software (Qiagen).

### 2.8. Statistical Analysis

Values were reported as mean ± standard deviation (SD). The Student’s *t*-test was used to compare the quantitative continuous variables. A *p* value < 0.05 was considered statistically significant. The statistical analysis was performed by GraphPad PRISM 5 software.

## 3. Results

In order to compare potential mutations between plasma-derived EV and cancer tissue in patients with glioblastoma, we analyzed a total of 26 subjects. Blood was withdrawn before surgery. Patients’ characteristics and inclusion criteria are described in Table 1 and methods, respectively. The mean age was 60.1 years, the male/female ratio was 1.4/1, and the mean pre-operative KPS was 89.5 ± 16.8%. Ten subjects were used as references for blood-derived EVs with a mean age 42 ± 11 years and a male/female ratio of 1/1.5 and were referred to the hospital for headaches.

In the recruited set of patients, the mean post-operative KPS was 91 ± 15.7%) (*p* > 0.05). Gross-total removal was reported in 12 (46.2%), near-total in 11 (42.3%), partial removal in 0 (0%), and biopsy was performed in 3 (11.5%). All patients underwent post-surgical adjuvant therapies according to Stupp et al. [4]. The mean recurrence-free survival was 13.2 ± 9.5 months, and the mean overall survival was 15.9 ± 9.72 months. The mean solid lesion volume calculated on gh-t1-weighted sequences was 16.1 ± 18.7 cm^3^. The mean necrotic lesion volume calculated on gh-t1-weighted sequences was 6.63 ± 16.11 cm^3^. The total volume measurement on gh-t1 weighted sequences was 23.45 ± 30.6 cm^3^, while the mean volume of the infiltrating non-enhancing component, calculated on FLAIR images, was 63.4 ± 56.6 cm^3^, thus meaning there was a solid-to-infiltrative volume ratio of 17.7%.

All histopathological features for the main diagnostic markers are reported in Table 2.

Out of 26 patients recruited, only the 20 patients with grade 4 glioblastoma were sorted.

**Figure 1:**
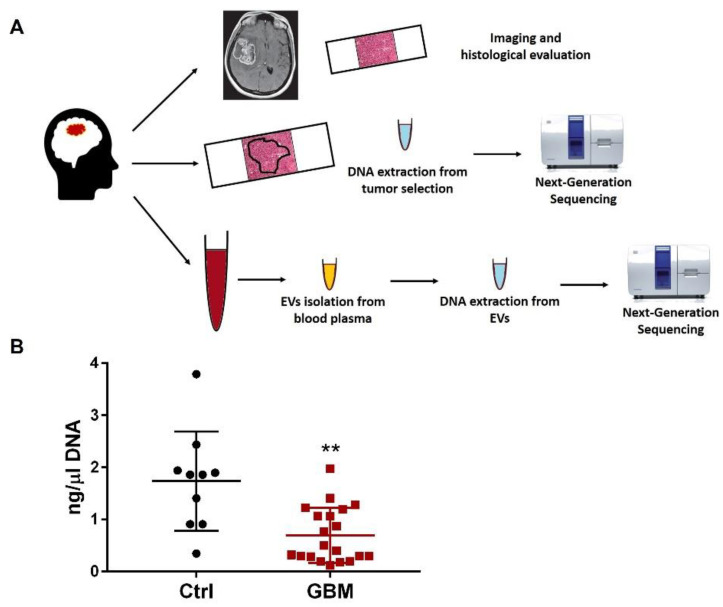
(**A**) Experimental workflow of the study. Brain tumor imaging and histopathological analysis were performed for cancer staging. A peripheral blood sample was also obtained from patients to isolate EVs. Afterwards, DNA was isolated in parallel from both paraffin-embedded sections and matched EVs and genomic mutations assessed by NGS analysis. (**B**) Blood-plasma-derived EV DNA concentration yield in patients with glioblastoma (*n* = 20) compared to healthy donors (*n* = 10). ** *p* < 0.01. GBM, patients with glioblastoma, Ctrl, controls.

Next-Generation Sequencing analysis was performed in parallel on both peripheral-blood-derived EVs and on the selected neoplastic area of the matched paraffin-embedded sections as depicted in the experimental plan (Figure 1A).

We performed the NGS analysis including the following genes: ATRX, CDKN2A, H3F3A, IDH1, IDH2, NF1, PTEN, TERT, and TP53. The genomic sequencing was performed only in samples (*n* = 10 patients and *n* = 5 controls; patients’ clinical features are described in Table 3) where we achieved high DNA standards in terms of quality and quantity (DNA threshold >0.5 ng/µL). Accordingly, the results showed a significantly lower DNA concentration contained in EVs isolated from plasma of patients with grade 4 GBM compared to controls (Figure 1B, *p* < 0.01).

**Figure 2:**
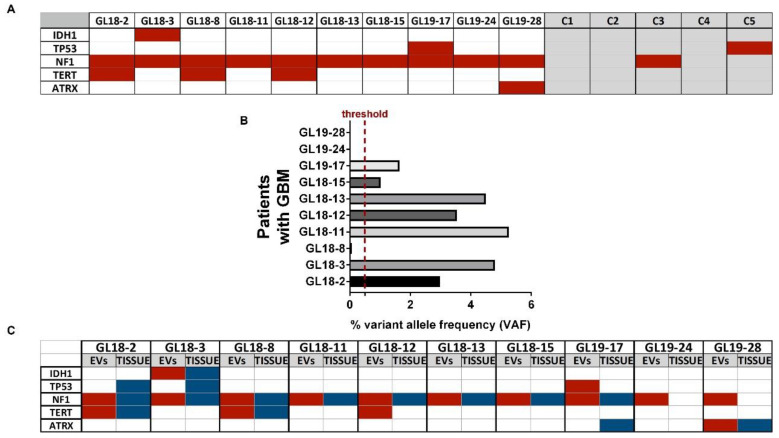
(**A**) Comparative analysis of pathogenic mutations (highlighted in red) found in peripheral-blood-derived EVs of patients with glioblastoma (*n* = 10) and healthy donors (*n* = 5). Acronyms GL18-2/19-28 and C1-C5 indicate patients and controls, respectively. (**B**) Variant allele frequency (VAF, expressed as percentage) of the pathogenic variant c.2568C>G of the NF1 gene in blood-derived EV of patients (*n* = 10). A >0.5% VAF cut-off threshold was set. Results are expressed as the mean ± SD. (**C**) Comparative analysis of pathogenic mutations (highlighted in red) found in peripheral-blood-derived EV and matched cancer tissue (highlighted in blue) of patients with GBM (*n* = 10). Acronyms GL18-2/19-28 and C1-C5 indicate patients and controls, respectively.

Interestingly, the mutational analysis by NGS on the same blood-derived EV samples has revealed that all patients displayed pathogenic mutations of the NF1 gene (10/10 patients) and that mutations of TERT were also moderately represented (3/10 patients, Figure 2A). Oppositely, peripheral-blood-derived EV samples from controls showed a homogenous wild-type genomic profile (Figure 2A). The results also highlighted that out of 10 patients, 7 exhibited the specific pathogenic variant of NF1 c.2568C>G corresponding to the p.S856R in blood-derived EVs with a variant allele frequency (VAF) >0.5% (Figure 2B). According to the Wikipathway database, the pathway affected was related to the MAPK signaling (https://www.wikipathways.org/index.php/Pathway:WP382 (accessed on 10 October 2022)).

The full list of the genomic variants classified as pathogenic that we found is reported in Table 4.

When the NGS analysis was performed in parallel on the corresponding cancer tissue, we observed that the mutational status of NF1 and TERT in the EV correlated with that from matched tumor tissue (8/10 and 2/10 patients for NF1 and TERT, respectively; see Figure 2C).

## 4. Discussion

To date, the development of novel biological and non-invasive markers is urgently required for all tumors but mainly for those as GBM challenged by the anatomical localization. Furthermore, the biology of GBM is poorly understood, hence there is still an increasing need to find novel markers to help classify this tumor, to follow its progression and to drive therapy.

From a diagnostic standpoint, extracellular vesicles are contextualized in the scope of the liquid biopsy. The rich cargo of nucleic acid, including DNA, is a very useful tool that can be exploited to reveal insights of the progression of the disease, as well as to understand the potential evolution of the genomic mutations caused by clinical treatments which are known to induce resistance and accelerate aggressiveness over the time. Accordingly, GBM is well-acknowledged to resist conventional therapies due to multiple mechanisms spanning from stem cell-like features to immunosuppression [26,27,28,29] and to secrete a very high number of EV [30]. Moreover, EVs can be released at any stage of cancer, exerting multiple biological functions including the modulation of oxidative stress [31,32], and carrying a DNA cargo more stable than the circulating free form due to the protection exerted by the cell membrane. This represents an important advantage as the genomic sequencing technique requires the integrity of the DNA, although potential contamination may arise from multiple cell sources when free nucleic acids are isolated from the blood [33].

In our preliminary study, we have already observed a correlation between the DNA content of blood-derived EVs obtained from patients with glioma and both tumor volume and mitotic activity [34]. In this report, we have increased the number of observations with specific focus on grade IV GBM, the most aggressive brain tumor.

Although it is acknowledged that patients with GBM exhibit an enrichment of EVs [35], insights into their DNA cargo are currently considered more critical to follow the evolution of a very heterogenous and dynamic tumor. The number itself of circulating EVs in patients with GBM has its own diagnostic significance during the follow up of patients, but it is subjected to profound variations post-surgery and chemotherapy or metastasis [36], strengthening the role of the molecular analysis of the cargo with respect to the sole quantitative analysis of EV. Hence, we found a decreased content of DNA in circulating EVs derived from our set of patients compared to controls. This result is quite in line with a previous publication showing that the amount of DNA localized inside the GBM-cell-derived EVs is lower than the DNA on the outer membrane [37], although other reports have highlighted that cancer exosomes, including GBM, carry a higher amount of DNA [38,39]. These discrepancies may originate from the heterogeneity and diversity of the DNA content in circulating EVs and from the different methodology of isolation.

Notably, we also found evidence for coherence between the genomic mutational profile of EVs and that found in matched tissue samples, suggesting that the DNA-based cargo of circulating EVs might be equally useful to reflect the profound intra-heterogeneity of GBM [40,41], allowing a potential quantitative and qualitative characterization of the tumor genome. To the best of our knowledge, only a couple of studies report the mutational analysis of genomic DNA isolated from peripheral-blood-derived EVs of patients with GBM [37,42].

Generally, the mutational analysis of EVs from patients with GBM refers to a panel of well-recognized mutations, including IDH1 and EGFR [42,43,44]. In our study, we have focused on additional genes of current interest in GBM [45]. We observed a homogenous tendency of all patients to display pathogenic mutations of the NF1 gene in circulating EVs as in matched cancer tissue. In other studies, mutations of NF1 are the expression of a subpopulation of the tumor [37]. Notably, NF1 has been recently discovered as a negative regulator of RAS/MAK signaling and controls the mesenchymal signature in GBM [46]. This is a key finding, as the difference of contribution between cancer cells and tumor microenvironment (also represented by EVs) has not clearly been elucidated yet in GBM. In line with this article, we have found that the most representative pathogenic genomic variant of NF1 was the c.2568C>G, which has been described only in liver neoplasm and affects the MAPK signaling pathway, whose role is acknowledged in tumor cell proliferation. We also found additional pathogenic variants that so far have been described for other types of cancers and that are required to be fully verified in GBM.

A prognostic value of circulating microparticles in patients with glioblastoma has been suggested [47], strengthened also by key observations including the transformation of astrocytes and the horizontal transferring of nucleic acid within the tumor microenvironment, resulting in the proliferation of the cancer counterpart [48]. Based on the biological role of EVs in GBM, the tumor progression index (TPI) is currently proposed as the novel predictive marker to include the molecular signature of the EVs, allowing a better distinction between cancer cells and healthy tissue [49]. Thus, it is conceivable that additional pathogenic genetic alterations will be included in the future.

To date, additional techniques such as FISH and immunohistochemistry are currently employed beyond the NGS, where often they are reported as discordant. Despite this, the high sensitivity and specificity of NGS still offers a versatile strategy to obtain an accurate landscape of useful markers for diagnostic purposes [50]. In fact, more genes can be simultaneously tested by NGS, and several panels of gene can be designed according to different clinical and research needs [51]. The gold standard for gliomas is to maximally integrate such techniques to accurately define the diagnosis and the therapeutic choice.

This study has several limitations. To reach a suitable pro-diagnostic validation of circulating EV in GBM to potentially correlate the result with the clinical parameters of the patients, a higher sample size is required. More importantly, we have not followed up with patients; therefore, it will be important to investigate potential changes in the molecular cargo of the DNA contained in the EVs in parallel to a more profound biological stratification of the patients and subtypes of GBM, a frequent phenomenon occurring in the same tissue area of this type of tumor. Notably, the NGS assessment was performed on the TERT gene rather than the promoter region. In fact, non-coding mutations of this latter are considered key drivers described with high frequency and representing the gold standard to characterize glioblastoma [52,53,54].

This study demonstrates the technical feasibility of the mutational analysis of the genomic cargo in circulating EVs and its utility as a clinical biomarker.

## Figures and Tables

**Table 1:** Baseline characteristics of the adult glioma cohort enrolled in the study.

Characteristics	Patients (*n* = 26)
Median age, years	60.1 (±16)
Gender (%)	
Male	57.7
Female	42.3
Tumor location (%)	
Frontal lobe	30.8
Right temporal lobe	11.5
Left temporal lobe	19.2
Temporo-parietal lobe	3.9
Temporo-insular	3.9
Occipital	11.5
Frontoparietal lobe	7.7
Parietal	11.5
Histology (WHO 2016) (%)	
IV	77.0
III	11.5
II	11.5
I	0
Surgical resection (%)	
Gross total	46.2
Near total	42.3
Partial	0
Biopsy	11.5
Median tumor volume, cm^3^ (±SD)	
Total Flair	63.4 (±56.3)
Non-enhancing (t1 Post-contrast)	6.6 (±16.8)
Enhancing (t1 Post-contrast)	16.1 (±18.7)
Enhancing + Non-enhancing (t1 Post-	23.4 (±30.6)
contrast)	
Karnofsky Performance Status (KPS) (%) preoperative	
90\100	76.9
80\70	19.2
<60	3.9
Karnofsky Performance Status (KPS) (%) preoperative	
90\100	84.6
80\70	9.2
<60	11.5
Progression (%)	
Yes	80.8
No	19.2
Free survival, months	13.23 (±17.9)
Survival, months	15.92 (±16.11)

**Table 2:** Immunohistochemical features of the adult glioma cohort enrolled.

Immunohistochemical Characteristics	Patients (*n* = 26)
GFAP	23
OLIG2	15
ATRX	2
EGFR	7
IDH1	3
p53	19
ki67 (mean % all patients)	28
mitosis (mean over 10 HPF all patients)	22.5

**Table 3:** Baseline characteristics of the adult glioma cohort of 10 patients screened for NGS analysis.

Characteristics	Patients (*n* = 10)
Median age, years	64.6 (±16.06)
Gender (%)	
Male	70
Female	30
Tumor location, (%)	
Frontal lobe	20
Right temporal lobe	20
Left temporal lobe	20
Temporo-parietal lobe	10
Occipital	20
Frontoparietal lobe	10
Surgical resection, (%)	
Gross total	30
Near total	40
Partial	10
Biopsy	20
Median tumor volume, cm^3^ (±SD)	
Total Flair	85.3 (±5.55)
Non-enhancing (t1 Post-contrast)	12.26 (±25.66)
Enhancing (t1 Post-contrast)	17.26 (±25.66)
Enhancing + Non-enhancing (t1 Post-	31.32 (±34.67)
contrast)	
Karnofsky Performance Status (KPS), (%) Preoperative	
90\100	70
80\70	20
<60	10
Karnofsky Performance Status (KPS), (%) Preoperative	
90\100	70
80\70	20
<60	10
Progression (%)	
Yes	90
No	10
Free survival, months	8.2 (±11.37)
Survival, months	10.5 (±8.14)

**Table 4:** Genomic variants with pathogenic significance found in blood-derived EV of the 10 patients with grade 4 glioblastoma.

Gene	Nucleotide Change	Amino Acid Change
*NF1*	c.233delA c.1466A>G c.1658A>G c.2027delC c.2568C>G c.3033delA c.3859T>C c.2297T>C	p.Asn78fs p.Tyr489Cys p.His553Arg p.Pro678fs p.Ser856Arg p.Thr1013fs p.Phe1287Leu p.Ile766Thr
*IDH1*	c.395G>A	p.Arg132His
*TP53*	c.700T>A c.1146delA c.841G>T	p.Tyr234Asn p.Lys382fs p.Asp281Tyr
*ATRX*	c.1074delA c.2658_2659delGA	p.Lys358fs p.Glu886fs
*TERT*	c.336delC	p.Glu113fs

## Data Availability

The main data generated or analyzed in this study are included in this article. Details are available from the corresponding authors upon reasonable request.
